# The Potential for Mindfulness-Based Intervention in Workplace Mental Health Promotion: Results of a Randomized Controlled Trial

**DOI:** 10.1371/journal.pone.0138089

**Published:** 2015-09-14

**Authors:** Shu-Ling Huang, Ren-Hau Li, Feng-Ying Huang, Feng-Cheng Tang

**Affiliations:** 1 Department of Psychology, Chung-Shan Medical University, Taichung, Taiwan; 2 Room of Clinical Psychology, Chung-Shan Medical University Hospital, Taichung, Taiwan; 3 Department of Education, National Taipei University of Education, Taipei, Taiwan; 4 Department of Occupational Medicine, Changhua Christian Hospital, Changhua, Taiwan; 5 Department of Leisure Services Management, Chaoyang University of Technology, Taichung, Taiwan; University of Pennsylvania, UNITED STATES

## Abstract

**Objectives:**

This study aims to intensively evaluate the effectiveness of mindfulness-based intervention (MBI) on mental illness risks (including psychological distress, prolonged fatigue, and perceived stress) and job strain (job control and job demands) for employees with poor mental health.

**Methods:**

A longitudinal research design was adopted. In total, 144 participants were randomized to the intervention group or the control group. The intervention group participated in MBI for eight weeks. Measurements were collected for both groups at five time points: at pre-intervention (T1), at mid-intervention (T2), at the completion of intervention (T3), four weeks after intervention (T4), and eight weeks after intervention (T5). Data were analyzed according to the intention-to-treat principle. A linear mixed model with two levels was employed to analyze the repeated measurement data.

**Results:**

Compared with the control group, the intercepts (means at T3) for the intervention group were significantly lower on psychological distress, prolonged fatigue, and perceived stress when MBI was completed. Even with the demographic variables controlled, the positive effects remained. For growth rates of prolonged fatigue and perceived stress, participants in the intervention group showed a steeper decrease than did the participants in the control group. Regarding job strain, although the intercept (mean at T3) of job demands showed a significant decline when BMI was completed, the significance disappeared when the demographic variables were controlled. Moreover, the other results for job control and job demands did not show promising findings.

**Conclusion:**

As a workplace health promotion program, the MBI seems to have potential in improving mental illness risks for employees with poor mental health. However, there was insufficient evidence to support its effect on mitigating job strain. Further research on maintaining the positive effects on mental health for the long term and on developing innovative MBI to suit job strain are recommended.

**Trial Registration:**

ClinicalTrials.gov NCT02241070

## Introduction

A certain percentage of the world’s working population has mental health-related problems. An estimated 27% of the working-age population in Europe experienced at least one type of mental health problem in the past year [1]. Mental health problems affect not only an individual’s quality of life, but also his or her work capacity. Long-term mental health problems of employees may lead to individual disability and inflated expenses, such as increased absenteeism, reduced productivity, greater compensation claims and high medical costs [2–4]. Workplace health promotion is therefore needed to enhance workers’ mental health. Mental health is described by WHO as “a state of well-being in which the individual realizes his or her own abilities, can cope with the normal stresses of life, can work productively and fruitfully, and is able to make a contribution to his or her community.” Due to the multifaceted definition of mental health, in order to affirm the effectiveness of a mental health promotion program, appropriate targets of assessment are necessary.

Psychological distress is common in workers, with an estimated prevalence of about 20%-30% worldwide [5–7]. In addition, prolonged fatigue and perceived stress are the most often reported symptoms among workers [8]. Prolonged fatigue is not easily reversible, not task specific, and does not disappear after a period of rest [9]. Prolonged fatigue increases vulnerability to physical and psychological problems [10]. Long-term high levels of stress are a known risk in regard to psychological distress and physiological diseases [11]. To improve workers’ health, mental illness risks, such as psychological distress, prolonged fatigue, and perceived stress, need to be considered and countered.

Job strain is a notable issue in today’s workforce. In Taiwan, a national survey found that more than 14% of employees “often” or “always” found their work to be very stressful [12]. The association between work stress and health problems has been well described [13,14]. The job demand-control model, based on psychosocial characteristics of work [15], is one of the most popular contemporary models for describing work stress. The model operates with two main dimensions: control at work and psychological job demands. High work demands coupled with low job control are considered to constitute job strain. However, recent research further indicates that low job control may be a better predictor of adverse health outcomes than high job demands [13]. Job control and job demands therefore could be explored separately.

Mindfulness-Based Intervention (MBI) is derived from Eastern contemplative traditions. In an operational definition, mindfulness includes two core components: (1) self-regulating attention and being curious and open, and (2) accepting one’s experience at the present moment [16]. Over the past decades, previous studies regarding MBI have found impressive reductions in psychological morbidity and pain for clinical groups, and mitigation of stress and enhanced emotional well-being for nonclinical samples [16–18]. Recently, MBI has been applied to workplace mental health promotion as well. Healthcare workers were the most common subjects surveyed [19–23]. In general, these studies found that MBI was effective for psychological distress, stress, and burnout. However, most of them were carried out with a small sample size of 30–50. In addition, the results were not consistent when MBI was applied to the employees other than healthcare personnel [24–28]. Moreover, most of these previous studies collected assessment data twice or thrice, at pre- and post-intervention, which made it difficult to clarify the changes of the treatment effects in detail.

The present study therefore adopted intensive measurements at five time points to explore the effectiveness of MBI as a workplace health promotion program on mental illness risks (psychological distress, prolonged fatigue, and perceived stress) and job strain (job control and job demands) among industrial employees with a larger sample size, with and without demographic variables controlled.

## Methods

### The Participants and Recruitment

This study was one component of the 2011 Taiwan Workplace Mental Health Promotion Scheme. The study adopted a randomized controlled study design. Two large-scale manufacturing factories (Factory A and Factory B) were chosen for this study. All of the factories’ 3270 full-time employees were requested to fill in a questionnaire comprising questions regarding mental health (measured by the Chinese Health Questionnaire, CHQ) and job strain (measured by the Job Content Questionnaire, JCQ; more details are provided in the section on Measures). Of these employees, 2849 individuals were willing to complete the questionnaire. The screening procedure was carried out between June and July in 2011. A total of 431 employees (15.13%) were found to suffer from poor mental health, which was defined by exhibiting both psychological distress and job strain in this study. That is, these employees needed to fulfill all the criteria: in the top tertile of the distribution in the CHQ for psychological distress; in the bottom tertile for the subscale of job control and in the top tertile for the subscale of job demands in the JCQ for job strain. A letter of invitation with an introduction to MBI was sent to these 431 employees. Of these, 144 responded that they would be willing to take part voluntarily in the study: 47 in Factory A and 97 in Factory B.

These 144 workers completed a baseline assessment (T1) and were then randomly allocated into the intervention group (Group I) or the waiting-list control group (Group C). To balance the sample size between groups and reduce noise or variance in the data, block randomization with a block size of four (ICIC design) was adopted for the present study [29]. In Factory A, 24 participants were allocated into Group I and 23 participants were into Group C. In Factory B, 48 participants were in Group I and 49 in Group C. In total, there were 72 participants in Group I, with the others in Group C. After randomization, two well-trained research assistants notified each participant about the group he or she was allocated to. The participants in Group C (n = 72) were requested to attend MBI class after completing four other times of assessment. In Group I (n = 72), one MBI class in Factory A (n = 24) and two MBI classes in Factory B (n = 48) were carried out to ensure an adequate class size of less than 25 for the efficiency of the intervention. All the participants were additionally measured at four other time points: at mid-intervention (T2), at the completion of intervention (T3), four weeks after intervention (T4), and eight weeks after intervention (T5). All the measurements (T1–T5) were taken with an interval of four weeks between measurements. The research assistants distributed and collected the questionnaire. Data for this study were obtained between August and December in 2011.

### Ethical Approval

This study was conducted according to the Helsinki Declaration and was approved by the Institutional Review Board of the Changhua Christian Hospital in Taiwan (IRB serial number: 110606) on July 25, 2011. Written informed consent was obtained from all the participants involved in this study. Before the Taiwanese Human Subjects Research Act was issued on Dec 28, 2011, only clinical trials according to the definition of ICH-GCP [30] were required to complete both the IRB review and clinical trial registration in Taiwan. Our study was approved by IRB and was completed prior to Dec 28, 2011. This is the reason that it was not registered prior to the enrollment of the first participant. The protocol for the trial and supporting CONSORT checklist are available as supporting information (see S1 Protocol, S2 Protocol and S1 CONSORT Checklist). The authors confirm that all on-going and related trials for this intervention are registered in the ClinicalTrials.gov (NCT02241070).

### Sample Size

Some open-source tools, such as GLIMMPSE, are available for calculating power and sample size for a general linear multivariate model. Mean differences in each measurement time, rather than effect size, are required [31]. However, no previous research adopting the variables compatible with our study can be referred to. In addition, multiple continuous covariates were used in this study, whereas only one continuous covariate is allowed for GLIMMPSE. GLIMMPSE therefore seems not applicable to this study. Instead, according to calculations by G*Power software, with medium effect size *f* equal to 0.25, type one error of .05 and 90% power being set up, a sample size of 104 participants suffices to detect a difference of means in a 2×5 mixed design ANOVA. Considering about 20–35% attrition, the 144 participants we recruited in the present research should be sufficient.

### Intervention

The MBI program included two hours of mindfulness training weekly and forty-five minutes of homework practice every day for eight weeks. The participants in Group I were divided into three classes: one in Factory A and two in Factory B. The same leader and professional facilitator led the classes together in order to prevent the outcomes from being affected by the different experiences of the leader and professional facilitator. The leader was a mindfulness and vipassana meditation trainer who had practiced both types of meditation for more than two decades, and had completed mindfulness trainer education; the professional facilitator was a mindfulness practitioner and a psychiatrist. The leader was also one of the authors, to ensure the intervention provided conformed to the curriculum structure. To prevent the classes from being influenced by the leader’s subjective bias, the data were kept from the leader during the period of data collection.

Each class met weekly for two hours in-session during paid working hours at the workplace. This 8-week MBI curriculum was based on the Mindfulness-Based Stress Reduction (MBSR) curriculum structure developed in the Stress Reduction Clinic of the University of Massachusetts Medical School, but without an all-day class [32]. However, the contents of the all-day class, such as silent meditation, mountain meditation, and loving-kindness meditation, were introduced in the sessions during the eight-week course. An overview of the MBI program in this study is available as supporting information (see S3 Protocol). In general, the mindfulness training followed the template MBSR program as much as possible. The Taiwanese version of home practice CD also followed the original version developed by Kabat-Zinn [32].

A number of mindfulness techniques, including both “formal” and “informal” practices with integration in the context of Mind/Body meditation, were explored in order to cultivate moment-by-moment present awareness with non-judgmental acceptance. The awareness is also open to every single experience, regardless of whether the experience is pleasant, neutral, or unpleasant. In addition, class discussions on sharing the experiences of using the mindfulness techniques and exploring how home practices and newly acquired mindfulness skills may be used correctly were encouraged in each session. The most important aspects of the class were the leader’s and facilitator’s demonstrations of how to live mindfully every day, equipping participants to develop their own mindfulness practice independently, and identifying whether participants used problematic or maladapted strategies to face or react to challenging situations, including facing their job-related problems [33,34].

### Measures

#### For mental illness risks

Psychological distress was measured by the Chinese Health Questionnaire (CHQ-12), a well-validated instrument [35]. The CHQ-12 was adapted from the General Health Questionnaire [36] with culturally relevant modification. The twelve items in the questionnaire, including questions pertaining to depression, anxiety, sleep disturbance, somatic concerns, and interpersonal difficulties, were on a four-point Likert scale. A higher score indicated a high level of psychological distress. For this study, alpha reliability was 0.83.

Fatigue is a common human experience. However, no gold standard or perfect definition exists for fatigue. Lewis and Wessely [37] noted several characteristics for fatigue: a subjective sensation of tiredness; having emotional, behavioral, and cognitive components; and being best viewed as a continuum. The Checklist Individual Strength questionnaire (CIS) [9] was developed to assess several aspects of prolonged fatigue: subjective fatigue, reduction in motivation, reduction in activity, and reduction in concentration; these are in line with the views of Lewis and Wessely [37]. The multidimensional CIS, which confirmed discriminant validity and convergent validity, was therefore used to measure prolonged fatigue in this study. The CIS consists of 20 items on a 7-point Likert scale [9]. The items refer to aspects of fatigue experienced during the previous 2 weeks. A higher score represented a higher level of prolonged fatigue. Cronbach’s alpha coefficient was 0.88 in the present study.

The 10-item Perceived Stress Scale (PSS-10) developed by Cohen and his colleagues was adopted to measure a global level of perceived stress [38]. The Chinese version of the PSS-10 demonstrated an adequate reliability and validity when used for a Chinese population [39]. In the present study, Cronbach’s alpha coefficient was 0.85. Participants were asked to respond to each question on a 5-point Likert scale ranging from 0 (never) to 4 (very often). A higher total score indicated a higher level of uncontrollable, unpredictable, and overwhelming feelings.

#### For job strain

The Job Content Questionnaire (JCQ) [40] is a commonly used questionnaire to assess job control (including 6 items pertaining to skill discretion and 3 items pertaining to decision authority), job demands (5 items), and workplace social support (8 items). The Chinese version of the JCQ [41] translated from the JCQ was reported to have acceptable psychometric properties and was adopted in this study. For the purpose of this study, only the subscales of job control and job demands were obtained. For each subscale, a sum of weighted-item scores was calculated according to the JCQ User’s Guide [40]. A higher-sum score represented a higher level of job control or job demands. In this sample, Cronbach’s alpha coefficients were 0.73 for the subscale of job control and 0.77 for the subscale of job demands.

### Statistical Analysis

Data were analyzed according to the intention-to-treat principle. All participants were analyzed according to the condition (Group I or Group C) they were initially randomized. Multiple imputation by SPSS 22.0 software package was adopted for missing data. The percentages of the personal characteristics of the participants were described, and the comparisons between Group I and Group C were conducted using the chi-square test. The comparisons of unadjusted data of the dependent variables between Group I and Group C at each time point were carried out by t-test. The linear mixed model with two levels combined was employed to analyze the repeated measurement data. Level 1 was an intra-participant level and level 2 was an inter-participant level. Level 1 included only a time predictor to obtain random intercept and slope terms as outcome variables at level 2 were predicted by only intervention for Model 1, or by intervention and some demographic variables (including gender, age, education, and occupation) for Model 2. We presented the analysis results with two models for each dependent variable. In Model 1, randomly varying intercept and slope (growth rate) were explained with only the intervention variable. Due to demographic variables possibly affecting an individual’s mental health, the demographic variables in level 2 were therefore controlled in Model 2. It is worthy noting that the time variable, including observations 1, 2, 3, 4, 5, was centered at median (T3) to form -2, -1, 0, 1, 2, so that the intercept term represented dependent variables at the third time measure (T3, completion of the intervention), not at the T1. The alpha level for statistical significance was set as 0.05. For easy application to workplace mental health promotion, occupation was divided into two groups: “white-collar” or “blue-collar”. Women in gender and blue-collar in occupation were reference groups. All analyses were carried out using the SPSS 22.0 software package.

## Results

**Fig 1:**
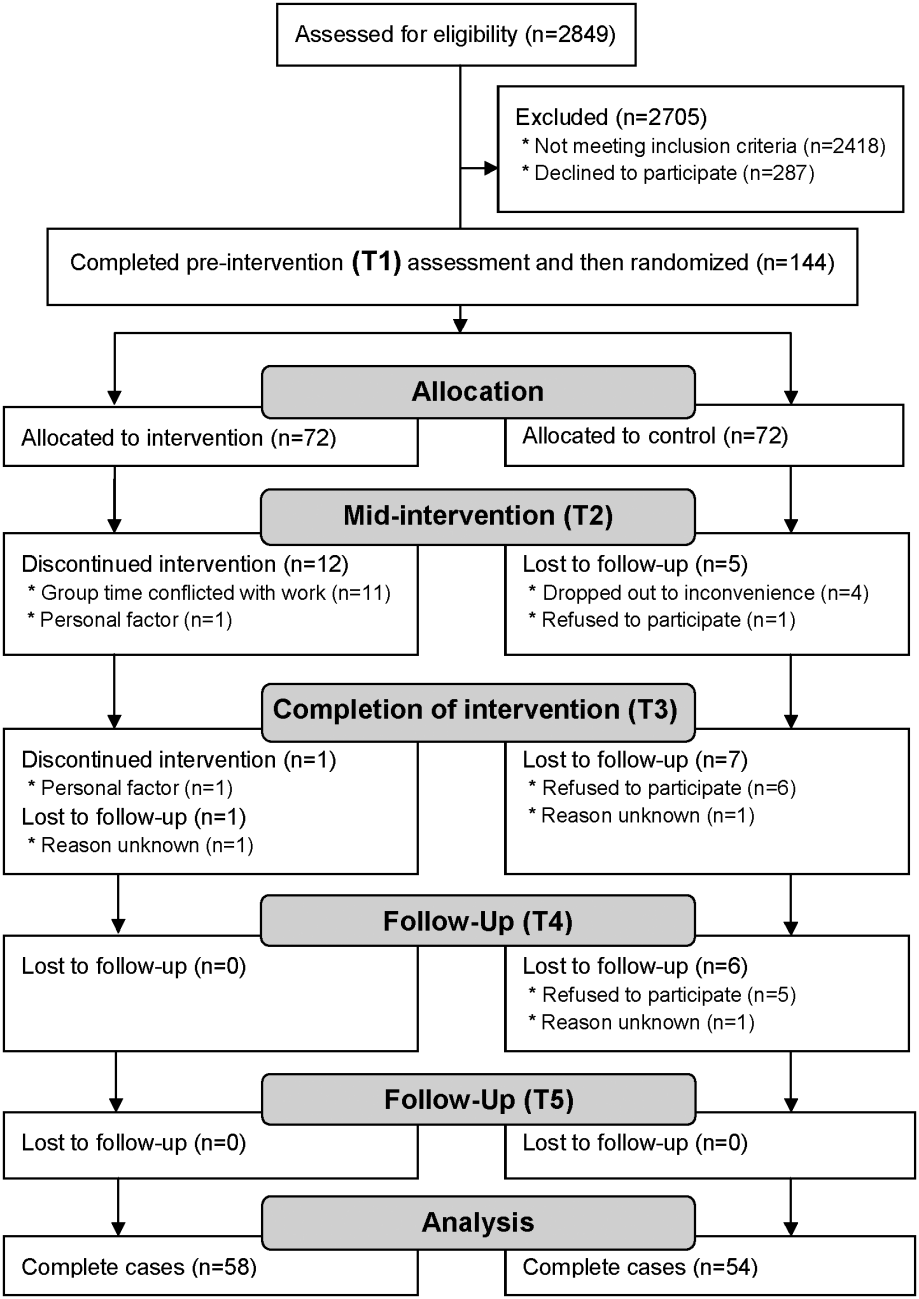
Flow diagram of MBI study.

One hundred and forty-four participants provided the initial and analyzed sample. The complete cases were made up of 58 participants in Group I (successful completion rate: 80.6%) and 54 participants in Group C (completion rate: 75.0%). The flow diagram of the study is presented in Fig 1. Table 1 shows the descriptive characteristics for the participants in Group I and Group C. The chi-square test revealed only statistically significant differences in gender between the groups [χ^2^ (1) = 4.85, *p* < .05]. The other characteristics did not show statistical significance between the groups.

**Table 1:** Comparing the characteristics of the participants in the intervention group (Group I) and control group (Group C).

Demographic variable	Group I (n = 72)	Group C (n = 72)	χ^2^ or t	p
Gender: Female, %	50.0	31.9	4.85	.028
Marital status: Married, %	73.6	70.8	0.14	.710
Education: College or above, %	83.3	80.6	0.19	.665
Occupation: White-collar, %	77.8	72.2	0.59	.441
Age, mean in year	42.4	42.7	-0.17	.863

After multiple imputing of missing data, Table 2 shows the comparisons of unadjusted data of mental illness risks (psychological distress, prolonged fatigue, and perceived stress) and job strain (job control and job demands) at each time point. No dependent variables at the baseline showed statistical difference between the groups. Psychological distress revealed difference at T2. At T3, all the results of mental illness risks and job strain showed significant differences between the two groups. The positive effects of MBI on mental illness risks (psychological distress, prolonged fatigue, and perceived stress) remained at T4 and at T5. In terms of job strain, after the MBI completion, only job demands showed a significant difference between the groups at T5.

**Table 2:** Comparisons of unadjusted data of the dependent variables between Group I (n = 72) and Group C (n = 72) at each time point.

		T1^#^			T2			T3			T4			T5		
	Group	Mean (SD)	t	p	Mean (SD)	t	p	Mean (SD)	t	p	Mean (SD)	t	p	Mean (SD)	t	p
Psychological distress			0.31	.759		-2.93	.004		-4.55	.000		-2.78	.006		-2.59	.010
	I	15.4(5.5)			10.8(6.0)			9.1(5.4)			10.3(5.6)			10.8(5.4)		
	C	15.1(5.4)			13.7(6.1)			13.7(6.7)			13.1(6.4)			13.3(6.2)		
Prolonged fatigue			-0.52	.604		-1.28	.203		-2.29	.023		-2.74	.007		-2.61	.010
	I	76.7(14.7)			73.3(15.6)			67.1(18.0)			67.5(17.8)			67.3(18.5)		
	C	78.0(13.9)			76.7(16.5)			74.2(19.2)			75.6(17.7)			75.6(19.2)		
Perceived stress			-1.60	.111		-1.08	.283		-2.85	.005		-2.92	.004		-2.83	.005
	I	19.8(4.3)			18.5(4.8)			17.3(5.4)			16.8(5.2)			16.2(5.4)		
	C	20.9(3.7)			19.4(4.7)			20.0(6.0)			19.4(5.4)			18.9(5.9)		
Job control			0.54	.592		0.05	.958		3.31	.001		1.78	.077		0.75	.456
	I	59.8(9.2)			61.8(7.9)			63.7(7.8)			62.3(9.2)			61.9(8.9)		
	C	59.0(8.3)			61.7(7.2)			59.1(8.7)			59.5(9.8)			60.7(9.8)		
Job demands			-1.00	.319		-0.11	.912		-3.27	.001		-1.89	.061		-2.15	.033
	I	32.3(5.6)			31.8(5.4)			29.0(5.3)			30.2(5.2)			30.0(5.3)		
	C	33.1(4.5)			31.9(4.8)			31.9(5.2)			32.1(6.8)			31.9(5.3)		

^#^Measured at pre-intervention (T1), at mid-intervention (T2), at the completion of intervention (T3), four weeks after intervention (T4), and eight weeks after intervention (T5).

The results of the linear mixed model on mental illness risks are summarized in Table 3. On psychological distress, Group I had significantly lower means than Group C did at T3 in Model 1 (β_01_ = -2.52, *p* = .001). In Model 2, after the demographic variables were controlled, the mean difference at T3 between Group I and Group C still reached statistical significance (β_01_ = -2.48, *p* = .001). For growth rate, the slope difference between Group I and Group C was not significant in Model 1 (β_11_ = -0.54, *p* = .056). However, in Model 2, with the demographic variables controlled, the growth rate for Group I was significantly lower than that for Group C (β_11_ = -0.67, *p* = .020).

**Table 3:** Fixed effects of Intervention to the intercept and growth rate for mental illness risks.

	Psychological distress				Prolonged fatigue				Perceived stress			
	Model 1		Model 2		Model 1		Model 2		Model 1		Model 2	
Fixed effect	Coefficient (S.E.)	*p*	Coefficient (S.E.)	*p*	Coefficient (S.E.)	*p*	Coefficient (S.E.)	*p*	Coefficient (S.E.)	*p*	Coefficient (S.E.)	*p*
For intercept,π_0i_												
Base,β_00_	13.78(0.53)	< .001	13.80(1.24)	< .001	76.00(1.65)	< .001	78.85(3.81)	< .001	19.71(0.49)	< .001	19.87(1.13)	< .001
Intervention,β_01_	-2.52(0.74)	.001	-2.48(0.76)	.001	-5.62(2.34)	.018	-5.59(2.34)	.018	-1.98(0.69)	.005	-1.97(0.70)	.005
Gender,β_02_			0.27(0.87)	.757			-0.65(2.68)	.808			0.19(0.80)	.813
Age,β_03_			0.01(0.05)	.815			-0.37(0.15)	.013			-0.10(0.04)	.018
Education,β_04_			-0.65(0.78)	.406			1.15(2.39)	.633			-0.39(0.71)	.589
Occupation,β_05_			-0.33(1.01)	.742			-3.17(3.10)	.309			-0.41(0.92)	.659
For growth rate,π_1i_												
Time,β_10_	-0.43(0.20)	.030	0.36(0.46)	.430	-0.59(0.54)	.281	2.69(1.21)	.028	-0.41(0.16)	.012	0.18(0.37)	.639
Intervention×Time,β_11_	-0.54(0.28)	.056	-0.67(0.28)	.020	-1.86(0.77)	.016	-2.24(0.75)	.003	-0.49(0.23)	.033	-0.51(0.23)	.027
Gender×Time,β_12_			-0.86(0.32)	.008			-3.16(0.85)	< .001			-0.41(0.26)	.121
Age×Time,β_13_			0.02(0.02)	.198			0.11(0.05)	.017			0.02(0.01)	.216
Education×Time,β_14_			0.14(0.29)	.624			1.56(0.76)	.043			0.42(0.23)	.076
Occupation×Time,β_15_			-0.28(0.37)	.449			-1.46(0.99)	.142			-0.39(0.30)	.201

Note: Time variable was recoded as -2, -1, 0, 1, 2, for five measurement time points in order. Women in Gender, blue collar in Occupation, and control group in Intervention were reference groups. Age was centered at mean and Education was centered at the level of college. The level 1 included a time variable only as the predictor, and level 2 included Intervention, Gender, Age, Education, and Occupation as predictors. For simplicity, variance components of level 1 and level 2 were omitted in the Table.

Each model was combined with level 1 and level 2 equations. For level 1 equation: *Y*_*ti*_ = *π*_0i_ + *π*_1i_ *Time*_*ti*_ + *e*_*ti*_. For level 2 equation:

*π*_0i_ = *β*_00_ + *β*_01_ *Intervention*_*i*_ + *r*_0i_ and *π*_1i_ = *β*_10_ + *β*_11_ *Intervention*_*i*_ + *r*_1i_ in Model 1;

*π*_0i_ = *β*_00_ + *β*_01_ *Intervention*_*i*_ + *β*_02_ *Gender*_*i*_ + *β*_03_ *Age*_*i*_ + *β*_04_ *Education*_*i*_ + *β*_05_ *Occupation*_*i*_ + *r*_0i_ and

*π*_1i_ = *β*_10_ + *β*_11_ *Intervention*_*i*_ + *β*_12_ *Gender*_*i*_ + *β*_13_ *Age*_*i*_ + *β*_14_ *Education*_*i*_ + *β*_15_ *Occupation*_*i*_ + *r*_1i_ in Model 2.

On prolonged fatigue, Group I had significantly lower means than Group C did at T3 in both models (β_01_ = -5.62, *p* = .018 for Model 1 and β_01_ = -5.59, *p* = .018 for Model 2). The growth rates of prolonged fatigue for Group I, compared with Group C, also showed significant differences in both models (β_11_ = -1.86, *p* = .016 for Model 1 and β_11_ = -2.24, *p* = .003 for Model 2).

On perceived stress, Group I had significantly lower means than Group C did at T3 in both models (β_01_ = -1.98, *p* = .005 for Model 1 and β_01_ = -1.97, *p* = .005 for Model 2). The growth rates of perceived stress for Group I in both models also showed significant differences compared with Group C (β_11_ = -0.49, *p* = .033 for Model 1 and β_11_ = -0.51, *p* = .027 for Model 2).

The summary of the linear mixed model on job strain is shown in Table 4. On job control, no significant mean difference appeared between Group I and Group C at T3 in both models. The growth rates of job control for Group I, compared with Group C, also showed no significant difference in both models.

**Table 4:** Fixed effects of Intervention to the intercept and growth rate for job strain.

	Job control				Job demands			
	Model 1		Model 2		Model 1		Model 2	
Fixed effect	Coefficient (S.E.)	*p*	Coefficient (S.E.)	*p*	Coefficient (S.E.)	*p*	Coefficient (S.E.)	*p*
For intercept,π_0i_								
Base,β_00_	60.00(0.89)	< .001	58.60(2.04)	< .001	32.17(0.53)	< .001	32.10(1.25)	< .001
Intervention,β_01_	1.87(1.25)	.137	1.92(1.26)	.128	-1.52(0.75)	.043	-1.50(0.77)	.054
Gender,β_02_			0.40(1.43)	.782			0.07(0.88)	.936
Age,β_03_			0.21(0.08)	.009			0.00(0.05)	.919
Education,β_04_			0.14(1.28)	.913			0.14(0.79)	.855
Occupation,β_05_			1.53(1.66)	.358			0.04(1.02)	.970
For growth rate,π_1i_								
Time,β_10_	0.11(0.25)	.660	-1.68(0.55)	.003	-0.24(0.16)	.149	-0.01(0.38)	.980
Intervention×Time,β_11_	0.35(0.35)	.315	0.53(0.34)	.116	-0.39(0.23)	.093	-0.41(0.24)	.086
Gender×Time,β_12_			1.55(0.39)	< .001			-0.22(0.27)	.406
Age×Time,β_13_			-0.05(0.02)	.012			0.02(0.01)	.119
Education×Time,β_14_			-0.53(0.35)	.130			0.22(0.24)	.370
Occupation×Time,β_15_			0.99(0.45)	.029			-0.09(0.31)	.775

Note: Time variable was recoded as -2, -1, 0, 1, 2, for five measurement time points in order. Women in Gender, blue collar in Occupation, and control group in Intervention were reference groups. Age was centered at mean and Education was centered at the level of college. The level 1 included a time variable only as the predictor, and level 2 included Intervention, Gender, Age, Education, and Occupation as predictors. For simplicity, variance components of level 1 and level 2 were omitted in the Table.

Each model was combined with level 1 and level 2 equations. For level 1 equation: *Y*_*ti*_ = *π*_0i_ + *π*_1i_ *Time*_*ti*_ + *e*_*ti*_. For level 2 equation:

*π*_0i_ = *β*_00_ + *β*_01_ *Intervention*_*i*_ + *r*_0i_ and *π*_1i_ = *β*_10_ + *β*_11_ *Intervention*_*i*_ + *r*_1i_ in Model 1;

*π*_0i_ = *β*_00_ + *β*_01_ *Intervention*_*i*_ + *β*_02_ *Gender*_*i*_ + *β*_03_ *Age*_*i*_ + *β*_04_ *Education*_*i*_ + *β*_05_ *Occupation*_*i*_ + *r*_0i_ and

*π*_1i_ = *β*_10_ + *β*_11_ *Intervention*_*i*_ + *β*_12_ *Gender*_*i*_ + *β*_13_ *Age*_*i*_ + *β*_14_ *Education*_*i*_ + *β*_15_ *Occupation*_*i*_ + *r*_1i_ in Model 2.

Regarding job demands, Group I had significantly lower means than Group C did at T3 in Model 1 (β_01_ = -1.52, *p* = .043); however, no significant difference appeared for the growth rate between Group I and Group C. In Model 2 with the demographic variables controlled, the mean difference at T3 disappeared (β_01_ = -1.50, *p* = .054). The growth rate of job demands in Model 2 did not show significant difference between the two groups as well.

## Discussion

The present study adopted a randomized controlled study design to determine the effectiveness of MBI as a workplace health promotion program. Compared with the control group, the intervention effects on psychological distress, prolonged fatigue, and perceived stress were revealed when the intervention was completed (T3). Furthermore, the positive effects on prolonged fatigue and perceived stress were maintained with time. However, in general, the effects of MBI on job control and job demands were not statistically significant. These findings show that MBI has the potential to lessen the mental illness risks for the workers with poor mental health, but not for job strain.

The literature includes limited reports on the use of MBI to improve workers’ mental health. Most of the reports showed results similar to ours. Studies using MBI for healthcare personnel showed that MBI was an effective strategy not only for reducing burnout and perceived stress, but also for increasing patients’ satisfaction [19–23]. The qualitative findings also revealed that greater relaxation and patience, more self-care and self-acceptance, improved inner peace, compassion and joy, better focus, and fewer somatic symptoms were the reported benefits [42,43]. In addition, research using a shortened worksite MBI showed improvement in perceived stress, psychological distress, positive orientation to life, and sleep quality among working adults [20,23,44,45]. How may MBI enhance mental health? Through the practice of meditation, individuals can achieve a state of mindfulness, in which thoughts and feelings are observed as events in the mind; any negative feeling or thought then become decentralized as “an event.” This dispassionate state of self-observation might create a “space” between one’s perception and response, helping to enhance emotional well-being and mental health [17].

However, not all the studies agree with our positive results. A worksite mindfulness-related multi-component health promotion intervention found no effect on work engagement, mental health, need for recovery, and mindfulness after 6 and 12 months of intervention [24]. Some possibilities might explain the inconsistency between this and our findings. First, in the study done by van Berkel et al. [24], all the voluntary participants were enrolled, so the ceiling effect might be the reason why the dependent variables could not be improved. Evidence was found of psychologically healthy participants diluting the observed effects of worksite stress management training programs [46]. An effective workplace health-promoting program needs to be tailored to the workers’ physical, psychological, or social needs [47]. Our study thus targeted the participants with poor mental health, and MBI was adopted to enhance their mental health. Second, lack of effects in the study of van Berkel et al. [24] might be due to the intervention’s being run on the participants’ own time. Our intervention was implemented during paid working hours. In other studies with positive results, their MBIs were also delivered at the workplace during work hours [20,22,25]. Effective workplace health-promoting interventions need the organization’s commitment to facilitating the changes both for employees and their working conditions [47]. This is the same as the action strategy of creating supportive environments as set forth in the Ottawa Charter of Health Promotion [48]. Another reason may be the different follow-up time. Based on the results of our study, the immediate and short-term positive effects of MBI on mental illness risks were observed. Determining how to modify the program and extend the positive effect to a longer period would be a challenge in the future.

Job control and job demands did not show significant improvements with time in Group I. The MBI class took place in working hours, and job content did not change, which might create other demanding characteristics. It might be one of the reasons why MBI did not work out in this field. However, there is a paucity of research on the relations between the level of mindfulness and either job control or job demands. Actual explanations are still unknown. Previous studies using MBI to improve work-related factors focused mainly on burnout and job satisfaction. The findings generally showed beneficial results in decreasing burnout and enhancing job satisfaction [20,22,49,50]. However, when MBI was adopted as an intervention for the outcomes which are not emotion-related, such as work engagement, lifestyle behaviors, and blood inflammation markers; all the results did not show significant differences [24,26,27]. The traditional MBI seemed to take effect mainly based on emotions or feelings. Besides, previous studies which tried MBI as an auxiliary strategy in eating behaviors and smoking cessation achieved some favorable results [51,52]. In the future, MBI with some innovations to apply to work-related dimensions or to health behaviors are recommended.

Notably, there were significant effects of time on psychological distress, prolonged fatigue, and perceived stress, with or without demographic variables controlled. It implies that both groups improved on mental illness risks with time; the participants in Group C also reduced psychological distress, fatigue, and stress, although the participants in Group I decreased more sharply. A possible explanation for this is that the participants in Group C also felt being cared for by being invited to take part in assessment and completing the questionnaire regarding mental health every four weeks. It implies that other simple programs to enhance workers’ mental health are worth developing in addition to MBI. In addition, the demographic variables showed their own respective effects on dependent variables. Decades of evidence have consistently shown that mental health is not equally distributed across socio-economic strata or gender [53–55]. However, previous studies using MBI as an intervention in workplace health promotion did not consider the influences of these variables. The present study attests to the significance of demographic variables, such as gender, age, and educational level, on mental health when implementing MBI for workplace health promotion.

The major strength of this study is the use of a randomized controlled study design with intensive repeated measures. This reliable research design could show the changes of dependent variables in detail with time. Second, the measured variables were multifaceted, matching the variety of mental health facets. Third, the sample size of the intervention group was relatively large compared with previous randomized controlled trials that generally included approximately twenty participants in the intervention. These aspects may avoid some research errors. However, some limitations in the study arose as well. First, most of the participants were married, highly educated, and white-collar. The inference of the findings should be applied with caution. Second, the extent of home meditation practice was thought to be associated with the effects of the MBI [56]. The study did not quantify and analyze the extent of home practice. Third, the waiting-list control group was not active. Some nonspecific factors, such as therapists’ attention, social support, and positive expectancy, cannot be excluded. The improved outcomes may be partly attributed to these factors. In addition, the study was not conducted in a blind study design; such study design did not prevent research outcomes from being influenced by the expectations of the participants in Group I. Finally, although one of the authors being the leader of MBI could ensure that the study was conducted smoothly and the intervention conformed to the curriculum structure, such design may entail the possibility of a conflict of interest and cannot eliminate subjective bias on the part of the leader. Further studies adopting active control groups with a double-blind design are thus recommended.

## Supporting Information

S1 CONSORT Checklist CONSORT Checklist. (DOC)

S1 Dataset The dataset of this study. (XLS)

S1 Protocol Trial Protocol in Chinese. (DOC)

S2 Protocol Trial Protocol in English. (DOC)

S3 Protocol MBI program protocol. (DOC)
