# Supplementary material for: The Potential for Mindfulness-Based Intervention in Workplace Mental Health Promotion: Results of a Randomized Controlled Trial
Source: PLoS One. 2015 Sep 14;10(9):e0138089. doi: 10.1371/journal.pone.0138089 (PMC4569475; doi:10.1371/journal.pone.0138089)
Supplement: S1 Protocol — (DOC) [file pone.0138089.s003.doc]

職場心理健康促進方案之成效：隨機分派準實驗法縱貫性研究

| 摘要  國民健康局積極推動「健康職場」以提升員工的健康。近年來，職場壓力及過勞等問題逐漸受到社會重視，台灣推動職場心理健康促進方案刻不容緩。正念減壓療程已被證實應用於減緩臨床患者的疼痛、睡眠障礙、壓力反應等等，有良好的成效。然而，正念減壓療程應用於工作壓力因應的成效，尚未被系統性地評估。  本研究目的(1)探討正念減壓療程及焦點團體法應用在改善工作壓力及心理困擾的成效；(2)分析工作者的基本變項及職場特性在不同處置模式的適用性。本計畫預計採取隨機分派準實驗法縱貫性研究設計，由職場提供高工作壓力員工名單，經徵求受試者同意後作為研究對象。其中兩家職場依隨機方式分派至正念減壓組及等待正念控制組。另兩家亦依隨機方式分派至焦點團體組及等待焦點控制組。每組預計各收100人，共計400位工作者為研究對象。於健康促進方案實施前、中、後，收集縱貫性問卷資料共五次。  本研究問卷包含基本資料、心理健康相關問卷、工作特質量表、以及活動滿意度調查。資料將使用SPSS軟體13.0版進行資料分析。以重複量數(repeated measure)檢驗介入處置前後，情緒困擾及工作壓力量表得分是否有顯著差異。重複量數亦可搭配介入活動變項，探討介入處置的不同方法對員工心理健康的影響，以及兩者的交互作用情形，可以觀察到介入處置方式隨時間變化的效果。以描述型統計方法（t檢定、ANOVA或ANCOVA）分析工作者的基本變項及職場特性在不同處置模式的適用性，以及確認正念減壓療程及焦點團體法之成效。期望本研究結果未來可依職場之性質、工作者之特性，提供職場具體、適用的建議。  關鍵詞：職場健康促進、心理健康、工作壓力 |
| --- |

**職場心理健康促進方案之成效：隨機分派準實驗法縱貫性研究**

1. 研究計畫之背景

歐美國家的工作者約有20%的員工有情緒困擾的問題（Marchand, Demers & Durand, 2005）。台灣的調查也顯示17%工作者有嚴重的情緒困擾（Huang et al.）。國民健康局為營造健康的職場工作環境，積極推動「健康職場」以提升員工的健康。近年來，職場壓力及過勞等問題逐漸受到社會重視，根據國際衛生組織估計，憂鬱將是勞工無法工作的第二大原因。因此，台灣推動職場心理健康促進方案刻不容緩。

過去研究發現，個人層次處置方案對於員工的情緒（例如憂鬱、焦慮）有較佳的改善，但對職業特性的感受（例如工作滿意度、工作壓力）沒有顯著影響；目前現行的組織層次處置方案對於職場相關感受改善較佳，但效果並不顯著（Kawakami et al. 1997; Peters & Carlson 1999; Mino et al. 2006）。持續發展適切的、實用的、成效持久的處置方案，仍是未來職場心理健康促進須要努力的方向。

正念減壓療程（mindfulness based stress reduction, MBSR）是美國麻省大學醫學中心附屬「減壓門診」的Jon  Kabat-zinn 博士於1979 年所創立的(Kabat-Zinn, 2005)。其目的乃在教導個案運用自己內在的身心力量，為自己的身心健康積極地做一些他人無法替代的事——培育正念。「正念」(mindfulness)指的是「純粹地注意當下每一秒所顯露的身心經驗」。正念減壓療程，是連續八至十週，每週一次為時2至3小時的團體訓練課程，教導成員學習以及實際練習培育正念的方法，並參加如何以「正念」面對、處理生活中的壓力與自身疾病的討論。過去研究證實將正念減壓療程應用於臨床患者的疼痛、睡眠障礙、壓力反應、情緒困擾、免疫功能，皆有良好的成效（Witek-Janusek, et al. 2008; Carlson et al. 2004; Robinson et al. 2003）。然而，將正念減壓療程應用於職場工作者工作壓力因應的相關研究卻付之闕如，應值得嘗試並探討其成效。

壓力源處置方案採用焦點團體法，焦點團體法是質性研究中經常使用的一種方法。其特色是針對討論的議題能夠在短時間內，收集到大量的資料及訊息。並經由鼓勵團體成員的互動及討論，使得研究過程中，能自然的擴展探討範圍、以及接觸十分具體的面向(Krueger & Casey, 2009)。尤其對於一些具爭議性的議題，也容易激發團體成員的不同反應，以進行進一步的討論（胡幼慧 2008）。本研究採用壓力源處置方案，使員工有機會深入檢視自覺工作壓力及情緒困擾的來源，討論如何抒解工作壓力，並共同暫議可行的措施及改變的可能性。團體帶領者也將根據團體的進展及成員的共同需求，提供必要的抒解方法。本研究將進行焦點團體法應用於職場健康促進的有效性。

1. 研究計畫目的

本研究目的是：（1）探討正念減壓療程及焦點團體法應用在職場上，對於工作者工作壓力及心理困擾改善的程度。（2）分析工作者的基本變項（性別、年齡、職業別）及職場特性（產業類別、職場大小）在不同處置模式的適用性。

1. 研究方法

1. 研究設計及研究對象

本計畫預計採取準實驗法研究設計，由職場健康促進人員提供工作壓力高危險群的名單，經徵求受試者同意後作為研究對象。將AB兩職場研究對象依隨機方式分派至正念減壓組或等待正念控制組；CD兩職場的研究對象依隨機方式分派至焦點團體組或等待焦點控制組。每組預計收100人，每組開課人數則依當時同意參與之受試者人數而定。

2. 研究步驟及資料收集

由職場健康促進人員提供工作壓力高危險群的名單，不同職場依隨機方式分派至各組。在告知同意後，一一邀請研究對象參與本研究。介入方案皆於邀請時，請受試者做一次前測評估。介入方案皆採八週的活動設計。介入方案皆由該領域專家帶領。將針對所有研究對象進行每個月一次的問卷資料收集，共四次追蹤，活動主題及詳細流程說明如下表。

**介入課程**

| 週次 | 問卷收集 | 正念組活動內容 | 焦點團體內容 |
| --- | --- | --- | --- |
| 零 | V | 無 | 無 |
|  |  | 團體簡介、建立團體規約，吃葡萄乾練習，身體掃描，回家作業說明 | 團體簡介、說明團體目的，自我介紹，建立工作關係；建立團體規約。討論工作壓力定義。 |
|  |  | 回家練習經驗之分享與討論，想法與感覺的練習，坐式正念練習。 | 確認工作壓力來源，進行壓力紓解討論可行措施及策略。 |
|  |  | 看或聽的正念練習，回家練習經驗之分享與討論，身與心的脈動，瑜珈，行禪，及三分鐘呼吸空檔練習。 | 視需求提供壓力紓解技巧。若團體共同認為壓力源是屬於組織層次的問題，可經共識後，邀請相關單位之主管參加 |
|  | V | 呼吸、身體、聲音及想法正念練習，回家練習經驗之分享與討論，三分鐘呼吸空檔練習與討論，慈悲觀想。 | 視需求提供壓力紓解技巧。必要時，邀請公司相關單位之主管參加。 |
|  |  | 覺察困難經驗之正念練習：覺察呼吸、身體、聲音與想法，並帶進一件有困難之經驗，覺察對身體感受的連結反應，回家練習經驗之分享與討論，慈悲觀想。 | 視需求提供壓力紓解技巧。必要時，邀請公司相關單位之主管參加。 |
|  |  | 提醒結束團體之準備，回家練習經驗之分享與討論，想像練習，高山正念練習，慈悲觀想。 | 視需求提供壓力紓解技巧。監控壓力紓解措施之進展，並提醒結束團體之準備。必要時，邀請公司相關單位之主管參加。 |
|  |  | 覺察困難經驗之正念練習，回家練習經驗之分享與討論，壓力管理計畫表，慈悲觀想。 | 監控壓力紓解措施之進展，並對未完成之事宜提供具體建議，協助後續之進展。 |
|  | V | 回家練習經驗之分享與討論，團體回顧，如何繼續保持過去七周正念練習訓練，無撿擇正念練習，慈悲觀想，結束團體。 | 回顧團體歷程，結束團體。 |
| 十二 | V | 無 | 無 |
| 十六 | V | 無 | 無 |

3. 研究工具

本研究問卷內容包含基本資料(人口學及工作相關變項)、心理健康相關資料(包含中國人健康量表、自覺壓力量表、疲勞量表)、 工作特質量表 (Job content Questionnaire, 簡稱JCQ)。各量表皆具良好信度、效度。

心理困擾程度以中國人健康量表（Chinese Health Questionnaire，CHQ）所測得。CHQ是根據英國精神科醫師Goldberg (1978) 所編製的General Health Questionnaire -12 (GHQ-12)，加入台灣的文化相關的因素修訂而成。CHQ被運用在篩選台灣的社區民眾的心理困擾，被證實具有良好的信效度及良好的辨識力(Cheng & Williams, 1986; Chen et al., 2000)。此量表共12題，受試者依照近兩週來的真實狀況來勾選符合的程度，以Likert四點量尺評分，各為「一點也不」、「和平常差不多」、「比平常較覺得」、「比平常更覺得」，計分為1分、2分、3分、4分。得分越高，表示心理困擾的程度越高。

疲勞量表 (Checklist Individual Strength, CIS) 由Beurskens等人所發展(2000)，共包含四個向度：主觀的疲勞經驗、工作動機、注意力及活動力。以Likert七點量尺評分，從「極不同意」到「極同意」，計分為1-7分。總分愈高，疲勞程度愈高。

工作特質量表 (Job content Questionnaire, JCQ) 是由Karasek提出(1985)，認為工作壓力的是工作負荷量和工作控制的交互作用所致，在高度的負荷量和低工作控制情境下會形成最強烈的工作壓力。工作控制指的是工作的決策幅度，包括工作自主性（decision authority）和技能裁量權（skill discretion）兩要素。工作負荷量的核心要素是完成任務所需要的精神負擔、精神警覺，高度工作負荷量包括：工作需要快速地完成、需要很努力地工作、沒有足夠時間完成工作等所造成的心理壓迫感…等。以Likert四點量尺評分，各為「很不同意」、「不同意」、「同意」、「很同意」，計分為1-4分。得分越高，分別代表工作控制及工作負荷的程度越高。

自覺壓力量表 (Perceived Stress Scale, PSS) 採Likert式五點計分法(Cohen, et al, 1983)，共10題，每題計分為「從未如此」(0分)，「幾乎不曾」(1分)，「有時如此」(2分)，「常常如此」(3分)，「總是如此」(4分)；第4、5、7、8題為反向題計分，總分最高為40分，分數越高表示壓力越大。

4. 分析方法

本研究資料將使用SPSS軟體13.0版進行資料分析。以重複量數(repeated measure)檢驗介入處置前後，情緒困擾及工作壓力量表得分是否有顯著差異。重複量數亦可搭配介入活動變項，探討介入處置的不同方法對員工心理健康的影響，以及兩者的交互作用情形，可以觀察到各種介入處置方式隨時間變化的效果。以描述型統計方法（t檢定、ANOVA或ANCOVA）分析工作者的基本變項（性別、年齡、職業別）及職場特性（產業類別、職場大小）在不同處置模式的適用性。

1. 預計完成之工作項目及成果

(1) 經由探討正念減壓療程及焦點團體法應用在職場上降低工作者的工作壓力及心理困擾的效果，以確定心理健康促進方案之效能。

(2) 藉由分析工作者變項、職場特性分別在使用正念減壓療程及焦點團體法的效果。未來可依職場之性質、工作者之特性，提供職場具體、適用的建議。

1. 參考文獻

- 胡幼慧。質性研究--理論、方法及本土女性研究實例。台北：巨流圖書公司，2008。

- Beurskens AJ. Bültmann U. Kant I. Vercoulen JH. Bleijenberg G. Swaen GM. Fatigue among working people: validity of a questionnaire measure. Occupational & Environmental Medicine. 57(5):353-7, 2000.
- Carlson LE, Speca M, Patel KD, Goodey E. Mindfulness-based stress reduction in relation to quality of life, mood, symptoms of stress and levels of cortisol, dehydroepiandrosterone sulfate (DHEAS) and melatonin in breast and prostate cancer outpatients. Psychoneuroendocrinology 29(4):448-74, 2004.
- Chen CS, Tsang HY, Chong MY, Tang TC. Validation of the Chinese Health Questionnaire (CHQ-12) in community elders. Kaohsiung J Med Sci 2000; 16: 559-65.
- Cheng Y, Luh WM, Guo YL (2003) Reliability and validity of the Chinese version of the Job Content Questionnaire in Taiwanese workers. Int J Behav Med 10: 15-30.
- Cheng, T.A. & Williams, P. (1986). The design and development of a screening questionnaire (CHQ) for use in community studies of mental disorders in Taiwan. *Psychol Med, 16*, 415-422.
- Cohen, S., Kamarck, T., Mermelstein, R. (1983). A global measure of perceived stress. Journal of Health and Social Behavior, 24, 385-396.
- Giga SB, Cooper CL, Faragher B. The development of a framework for a comprehensive approach to stress management interventions at work. Int J Stress Manage. 10:280-296, 2003.
- Goldberg, D.P. (1978). *The manual of the general health questionnaire.* London: NFER-NELSON Publish.
- Huang SL, Lee HS, Li RH, Lai YM, Chen AL, et al. (in submission) Differences in health complaints among Taiwanese workers in different occupational categories. J Occup Health.
- Kabat-Zinn J (2005) Coming to our senses. New York: Hyperion.
- Karasek RA (1985) Job content questionnaire and user’s guide. Lowell: University of Massachusetts.
- Kawakami N, Araki S, Kawashima M, Masumoto T & Hayashi T. Effects of work-related stress reduction on depressive symptoms among Japanese blue-collar workers. Scand J Work Environ Health. 23(1):54-9, 1997.
- Krueger, R.A., & Casey, M.A. (2009). Focus groups: A practical guide for applied research (4th Ed.).Thousand Oaks, CA: Sage Publications.
- Marchand A, Demers A, Durand P (2005) Do occupation and work conditions really matter? A longitudinal analysis of psychological distress experiences among Canadian workers. Sociol Health Illn 27: 602-627.
- Mino Y, Babazono A, Tsuda T & Yasuda N. Can stress management at the workplace prevent depression? A randomized controlled trial. Psychother Psychosom. 75(3):177-82, 2006.
- Peters KK & Carlson JG. Worksite stress management with high-risk maintenance workers: a controlled study. Int J Stress Manage. 6(1):21-44, 1999.
- Robinson FP, Mathews HL, Witek-Janusek L. Psycho-endocrine-immune response to mindfulness-based stress reduction in individuals infected with the human immunodeficiency virus: a quasi-experimental study. J.Altern.Complement Med. 9(5):683-94, 2003.
- Witek-Janusek L, Albuquerque K, Chroniak KR, Chroniak C, Durazo-Arvizu R, Mathews HL. Effect of mindfulness based stress reduction on immune function, quality of life and coping in women newly diagnosed with early stage breast cancer. Brain Behav.Immun. 22(6):969-81, 2008.

附錄

附件，AF01-010/04.3

IRB初審案申請書

| 計畫編號 | | |  | | IRB編號 | | | 110606 | | | | |
| --- | --- | --- | --- | --- | --- | --- | --- | --- | --- | --- | --- | --- |
|  |  |  |  |  | 收件日期(由本會填寫) | | | ___年___月___日 | | | | |
| 計畫名稱 | 中文 | | 職場心理健康促進方案之成效：隨機控制組準實驗法 | | | | | | | | | |
|  | 英文 | | Effects of mindfulness based stress reduction and focus group on dealing with job stress and emotional disturbance: a longitudinal study with randomized quasi experimental design | | | | | | | | | |
| 研究成員 | | | 中文姓名 | 英文姓名 | | 電話/分機 | | | e-mail | | | |
| 主持人 | | | 湯豐誠 | Feng-Cheng Tang | | 04-7238595#4131 | | | 106159@cch.org.tw | | | |
| 協同主持人 | | | 黃淑玲 | Shu-Ling Huang | | 04-24730022#12301 | | | shuling@csmu.edu.tw | | | |
| 研究人員 | | | 李仁豪 | Ren-Hau Li | | 04-24730022 #11351 | | | davidrhlee@yahoo.com.tw | | | |
| 研究人員 | | | 黃鳳英 | Feng-Ying Huang | | 02-66396688# 55728 | | | fayin66h@gmail.com | | | |
| 研究人員 | | | 張梅香 | Mei-Hsiang Chang | | 0981-680353 | | | home2781454@yahoo.com.tw | | | |
| 研究人員 | | | 莊琇菱 | Hsiu-Ling Chuang | | 04-7238595/4131 | | | 396477@cch.org.tw | | | |
| 聯絡人  ■院內  □院外 | | | 湯豐誠 | Feng-Cheng Tang | | 04-7238595#4131 | | | 106159@cch.org.tw | | | |
| 經費贊助者 | | | - 無 | ■ 有： 行政院衛生署國民健康局 | | | | | | | | |
| 有無為受試者保險 | | | ■ 無 | - 有：__________________________________ | | | | | | | | |
| 相關文件 | | 計畫書 | | 受試者同意書 | 個案報告表 | | 主持人手冊 | | | | | 廣告文件 |
| 版本 | | 1.0 | | 1.0 | N/A | | N/A | | | | | N/A |
| 日期 | | 201105016 | | 20110516 | N/A | | N/A | | | | | N/A |
| 計畫執行期限 | | | 共約__1__年__0__月 | | | | | | | | | |
| 申請狀態 | | | ■ 簡易審查 | | □ 一般審查 | | | | | | - 其他：_______ | |
|  |  |  | - 本計劃與先前本會審查過的計畫案類似，IRB編號：   ■ 是否送其他IRB審查  □是。如已有審查結果，請檢附審查意見： 。  ■否 | | | | | | | | | |
| 地點類型 | | | ■ 單一中心  □本院  ■其他：4家相關職場 | | - 多中心   □國內  □跨國 | | | | | | 已建立多中心研究計畫之溝通管道  □是 □否 | |
| 若為藥品研究，請寫下列欄位：   1. 藥品名稱： 2. 主成分： 3. 劑型： 4. 劑量： 5. 製造廠、國別： 6. 同成份劑型、劑量藥品上市情形   國 內：上市日期 □查驗登記尚未申請 □查驗登記申請中  原 產 國：上市日期 □查驗登記尚未申請 □查驗登記申請中  其他國家：□查驗登記尚未申請 □查驗登記申請中   1. 本試驗用藥屬性  - 新藥：□新成份 □新使用途徑 □新複方 □新適應症 - 新劑型( □速效劑型 □持效性釋出劑型 □其他 ) - 新使用劑量 - 新單位含量 - 學名藥(監視期間) - 其他 | | | | | | | | | | | | |
| 研究型態 | | | - 藥品查驗登記 - 藥品學術研究 - 遺傳材料樣本 - 附加試驗 - 流行病學 - 前瞻性   ■ 非侵入性 | | - 新醫療技術 - 上市後監測 - 組織培養樣本 - 延伸試驗   ■ 問卷型   - 回溯性 - 其他：________ | | | | | | - 新醫療器材 - 基因研究 - 使用血液樣本 - 銜接試驗 - 記錄型 - 侵入性 | |
| 試驗階段 | | | - phase I：以了解藥物毒性為目的之安全性研究，對象為健康志願者。 - phase II：以了解藥物療效為目的之初步療效觀察，對象為病人。 - phase III：以確認療效及安全性為目的之完整療效評估，對象為病人及對照組。 - PhaseIIIa尚未通過主管機關審核。 - phaseIIIb已通過主管機關審核。 - phase IV：藥物上市後的安全性監視，對藥物是否產生不良反應，進行長期的追蹤。 - 其他： 。 | | | | | | | | | |
| 研究設計 | | | ■ 平行研究 | | - 交叉研究 | | | | | | ■ 其他：  問卷調查 | |
| 研究組數 | | | □ 單組 | | □ 雙組 | | | | | | ■ 多組： 4 組 | |
| 是否有 | | | ■ 對照組   - 安慰劑 - 有效藥 (藥品、劑型、用法)   ■ 其他 無參與課程活動之高壓力工作者 | | | | | | | | | |
|  |  |  | ■ 隨機分配 | | | | | | | | | |
|  |  |  | - 盲性 | | | | | | | | | |
|  |  |  | ■ 開放 | | - 單盲 | | | | | - 雙盲 | | |
|  |  |  | - 評估者盲性 | | - 其他 | | | | |  | | |
| 樣本數量 | | | - 全球_______人 | | ■ 國內 400 人 | | | | | - 本院_______人 | | |
| 試驗對象 | | | ■ 年齡範圍： 18 歲～ 65 歲 | | | | | | | | | |
|  |  |  | ■ 正常人   - 病人   ■ 男性  ■ 女性   - 其他：______ | | □易受傷害族群  □受刑人  □孕婦  □嬰兒  □兒童  □身心障礙者  □其他：________ | | | | | | | |
| 特殊條件 | | | - 加護照顧 - 孩童加護照顧 - 基因治療 - 義肢 | | - 隔離 - 靜脈注射 - 管制藥品 - 其他：________ | | | | | | - 手術 - 電腦斷層掃描 - 婦科用品 | |
| 納入條件 | | | 18-65歲全職工作者，有心理困擾及工作壓力者。  每組預計各篩選出100人，共計 400位職場工作者為研究對象。 | | | | | | | | | |
| 排除條件 | | | 小於18歲，大於65歲者。  非全職者。  沒有意願參加本計畫者、無法溝通理解者。 | | | | | | | | | |
| 預期不良事件/嚴重不良事件 | | | 本研究不涉及藥物使用及抽血，故無不良事件。 | | | | | | | | | |
| 預期不良事件/嚴重不良事件之處理方式 | | | 本研究不涉及藥物使用及抽血，故無不良事件。 | | | | | | | | | |
| 招募受試者方式 | | | - 媒體廣告（須檢附文件內容） - 電視 □廣播 - 報紙 □網路 - 電子看板 □海報 - 廣告單張 | | | | | | | | ■ 口頭介紹   - 其他________ | |
| 試驗結果之報告或發表方式 | | | 研究結果將繳交國民健康局成果報告，並發表於期刊或研討會。 | | | | | | | | | |
| 1. 對於易受傷害族群在取得知情同意過程中，是否有受到特別的保護？   □是，說明__________  □否  ■不適用 | | | | | | | | | | | | |
| 1. 是否設置資料安全監測計畫(Data safety monitoring plan; DSMP)   說明:除極小危險試驗外，計畫案皆須設置DSMP，極小危險試驗之定義：對身體或心理上造成的傷害的機會或程度，相當於健康受試者的日常 生活、常規醫學及心理學檢查所造成者，並沒有因為參與人體試驗而增加。  □是；關於設置DSMP，計畫書是否包含下列內容？(請勾選)  □試驗的風險等級  □監測的完整性  □AE的嚴重程度  □如何通報未預期AE  □如何通報年度安全性報告  □DSMP的監測者、監測方式及監測頻率  ■否 | | | | | | | | | | | | |
| 1. 是否設置資料安全監測委員會(Data safety monitoring board ; DSMB)   □是: (試驗符合下列狀況須設置DSMB，(請勾選)  □對照性研究，其主要或次要終點是死亡和/或發生嚴重疾病。  □隨機對照的研究，其主要的研究目的是評價降低嚴重疾病的發病率或死亡率的一項新干預措施的有效性與安全性。  □高風險干預措施的早期研究，無論是否隨機，例如︰有不可預防的、潛在致命的併發症風險，或常見的、引起關注的、可預防的不良事件。  □創新性的干預措施的早期階段研究，其臨床安全性訊息非常有限，或先前的資料引起有潛在嚴重不良後果的關注。  □設計複雜的研究，或預期累積的數據難以解釋的研究，或累積的數據可能影響研究的設計和受試者安全性問題的研究，尤其是長期的研究。  □研究中獲得的數據證明應中止研究，如一干預措施的目的在於降低嚴重疾病的發病率或死亡率，而事實可能有不良回應或缺乏療效，導致發病率或死亡率增加。  □在緊急情況下實施的研究。  □涉及弱勢人群的研究。  □多中心研究計畫案。（FDA）  繳交DSMB報告之頻率：_______________  ■否 | | | | | | | | | | | | |
| 1. 是否為試驗目的而取消或暫停標準治療？   □是，說明__________  □否  ■不適用 | | | | | | | | | | | | |
| 1. 是否將不能行使同意者納入試驗?   □是，理由__________  □否  ■不適用 | | | | | | | | | | | | |
| 1. 試驗期間及試驗後，是否提供受試者醫療照護？   □是  □否  ■不適用 | | | | | | | | | | | | |
| 1. 計畫結束後，是否有提供受試者繼續取得試驗產品之計畫？   □是  □否  ■不適用 | | | | | | | | | | | | |
| 1. 可能接觸受試者個人資料(包括其醫療記錄及檢體)之人員？   包括：_主持人、協同主持人及研究人員(不包含可辨識受試者身分之個人資料)。 | | | | | | | | | | | | |
| 1. 受試者資料保密方式：   ■ 受試者身分編碼 ⬜ 英文縮寫名識別 ■ 將研究資料編碼  ■ 所有資料上鎖/加密 ⬜ 其他： | | | | | | | | | | | | |
| 1. 未去連結之研究材料是否提供國外特定研究使用？   □是，應檢付國外研究執行機構可確保遵行我國相關規定及研究材料使用範圍之擔保書  ■否  (註：研究材料係指研究對象之人體檢體、自然人資料及其他有關之資料、資訊) | | | | | | | | | | | | |
| 1. 受試者同意書取得之相關程序：   是否申請免除或改變知情同意？ ⬜否 ■是(請檢附改變或免除知情同意檢核表)   - 1. 由誰向受試者或其法定代理人解釋研究內容並取得同意？   ⬜計畫主持人(含共/協同主持人)  ■計畫研究人員  ⬜其他醫師/研究護士 (非共/協同主持人)   - 1. 取得同意書人員使用之語言：   ⬜國語 ⬜台語 ■國語+台語 ⬜其他：   - 1. 預期納入之受試者或其法定代理人之可理解的語言   ⬜國語 ⬜台語 ■國語+台語 ⬜其他：   - 1. 取得同意的時機？   ⬜篩選前  ⬜篩選後，隨機分派前  ■篩選後，隨機分派後   - 1. 在什麼地點解釋試驗內容？ 受試者的職場 ；每件約花費多久時間？15-20 分鐘 | | | | | | | | | | | | |
| 1. 受試者之風險利益評估：    1. 實驗組：   ■ 相當於微小風險。(第一類風險)   - 超過微小風險，但對受試者有直接利益。(第二類風險) - 超過微小風險，但對受試者無直接利益，但有助於了解受試者之情況。 - 超過微小風險，且對受試者無直接利益，但研究主題可得到價值的結果。(第四類風險)   1. 對照組： ■有 □無(以下選項免填)   ■ 相當於微小風險。(第一類風險)   - 超過微小風險，但對受試者有直接利益。(第二類風險) - 超過微小風險，但對受試者無直接利益，但有助於了解受試者之情況。(第三類風險) - 超過微小風險，且對受試者無直接利益，但研究主題可得到價值的結果。(第四類風險) | | | | | | | | | | | | |
| 填寫人聲明 | | | 以上資料由本人負責填寫，已盡力確保內容正確。若有不實或蓄意隱瞞，願負法律上應負之責任。  填寫人簽名：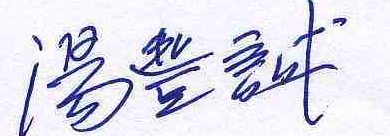  單位：_ 財團法人彰化基督教醫院  日期： 2011 年 5 月 16 日 | | | | | | | | | |
| 主持人聲明 | | | 1. 本人負責執行臨床試驗，已仔細閱讀過試驗計畫書，本人承諾將依貴人體試驗委員會（以下簡稱委員會）同意之試驗計畫書，進行試驗。 2. 本人明瞭並同意遵守赫爾辛基宣言、國內與人體試驗有關之倫理、法律及主管機關相關法令的要求，確保受試者之權利、安全、個人隱私以及福祉受到保護。 3. 依法令規定，委員會有權檢視任何與試驗相關之資料、進行實地訪查、追蹤審查經核准試驗之執行進度，且追蹤審查每年不得少於一次。本人承諾定當配合辦理，並如期繳交期中報告。 4. 若發生重大影響臨床試驗執行或增加受試者風險之情形時，本人應立即向委員會提出書面報告。 5. 受試者發生任何嚴重不良事件或發生未預期之嚴重不良事件時，本人應立即通知委員會及主管機關，並提供詳細書面資料。 6. 除為及時避免受試者遭受傷害外，在未獲得委員會同意前，本人絕不會偏離或變更試驗計畫書之執行。 7. 試驗完成或提早終止時，本人應向委員會提出試驗結果摘要。   主持人簽名：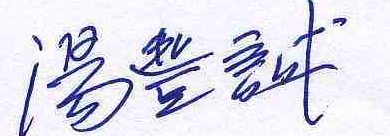  單位：_ 財團法人彰化基督教醫院  日期： 2011年 5 月 16 日 | | | | | | | | | |
