# Supplementary material for: The Potential for Mindfulness-Based Intervention in Workplace Mental Health Promotion: Results of a Randomized Controlled Trial
Source: PLoS One. 2015 Sep 14;10(9):e0138089. doi: 10.1371/journal.pone.0138089 (PMC4569475; doi:10.1371/journal.pone.0138089)
Supplement: S2 Protocol — (DOC) [file pone.0138089.s004.doc]

Effects of mindfulness based stress reduction and focus group on dealing with job stress and emotional disturbance: a longitudinal study with randomized quasi experimental design

| Abstract  Mindfulness-based intervention (MBI) is a newly psychotherapy program. It has been evidenced to be beneficial for decreasing pain, sleep problems, stress reactions, and emotional disturbance. Knowledge of the benefit of MBI to job stress is little. Focus group is a good way to obtain a great amount of relevant information regarding with the discussed issues in a short time. However, the systematic evaluation of the effects of Focus group applying at workplace is limited. The aims of the study are (1) to explore the effects of MBI and Focus group on job stress and workers’ psychological problems, (2) to examine the influence of workers’ characteristic (gender, age, and occupation) on the effects of MBI and Focus group.  The study will adopt a longitudinal research design with randomized quasi- experimental trial. Four companies are chosen for the study. The workers at high risk for mental distress provided by the company will be invited to take part in the study. The participants from two of four companies will be randomly assigned to MBI (Group M) or the waiting-mindfulness-list control group (Group C-M). The participants from the other two companies will be randomly assigned to Focus group (Group F) or the waiting–focus-list control group (Group C-F). One hundred members will be recruited in each group. Longitudinal Data will be collected five times at pre-, during, post- the intervention and follow-up twice with a four-week interval between measurements.  The research questionnaire will include basic information, Chinese Health Questionnaire, Checklist Individual Strength Questionnaire, and Perceived Stress Scale, Job content Questionnaire. All the questionnaires are validated instruments. SPSS-13.0 will be used for statistical analysis. Repeated measure will be adopted to examine the effects between pre- and post- interventions on job stress and emotional disturbance. The comparisons between the intervention group (Group I) and the waiting-list control group (Group C) will be conducted using the chi-square test or Student’s t-test. The general linear model will incorporate repeated measures: a two-way mixed design ANOVA will be performed to determine the interactive effects and then the simple main effects of intervention and the five continuous time points for the measured dependent variables. In addition, in order to explore the influences of personal characteristics on the outcomes of the intervention with time, the interaction and simple main effects of time by gender, age, education, and occupation on the measured dependent variables will be analyzed as well.  Keywords: workplace health program; mental health; job stress |
| --- |

**Effects of mindfulness based stress reduction and focus group on dealing with job stress and emotional disturbance: a longitudinal study with randomized quasi experimental design**

**Introduction**

A certain level of the world’s working population has mental health-related problems. About 19% of workers in Canada had reported more than one episode of psychological distress (Marchand, Demers & Durand, 2005). In Taiwan, a cross-sectional study showed that an estimated 17% of workers suffered from serious psychological distress (Huang et al.). Action in this field should therefore be launched more aggressively. Workplace health promotion program was found to be effective on enhancing workers’ mental health (Kawakami et al. 1997; Peters & Carlson 1999; Mino et al. 2006).

Mindfulness-Based Intervention (MBI) is derived from Eastern contemplative traditions. Mindfulness involves at least five characteristics: non-reactivity to inner or external experience; observing sensations, perceptions, thoughts, and feelings with non-conditional acceptance; acting with awareness; describing sensation, emotion, and thoughts with words; and non-judging of experience (Kabat-Zinn, 2005). For the past decades, the previous studies regarding MBI have found impressive reductions in psychological morbidity and pain for the clinical groups and the mitigation of stress and enhanced emotional well-being for nonclinical samples (Witek-Janusek, et al., 2008; Carlson et al., 2004; Robinson et al., 2003). MBI was seldom applied to workplace mental health promotion; its effects on mental health for workers are worth investigating.

Focus group is defined as "carefully planned series of discussions designed to obtain perceptions on a defined area of interest in a permissive, nonthreatening environment.” (Krueger & Casey, 2009) Focus groups are a way to get information about attitudes, beliefs, feelings and emotional reactions. It is also a good method to get both individual and interactive opinions (Hwu, 2008). Focus groups can be used for program development or to evaluate an existing program or intervention. It seems to be an appropriate strategy for assessing job strain among workers. However, the systematic evaluation of the effects of Focus group applying at workplace is limited.

**Purpose of the study**

The aims of the study are to determine the effectiveness of MBI and Focus group as a workplace health promotion program on psychological distress, prolonged fatigue, job strain (job control and job demand), and perceived stress and to explore the influences of personal characteristics (including gender, age, education, and occupation) on the outcomes of each intervention with time.

**Method**

Study design and the participant

The study will adopt a randomized controlled study design. Four manufacturing factories are chosen for this study. All the full-time workers in the factories will be requested to fill out a questionnaire regarding mental health and job content. The screening procedure was approved by the Institutional Review Board of the Changhua Christian Hospital. After pre-interventions assessment, the workers with psychological distress and job strain would be invited to participant in the study. The workers who are willing to take part in the study will need to give their informed consent and will be the participants for the study. The participants from two of four companies would be allocated into the MBI group (Group M) or the waiting-mindfulness-list control group (Group C-M) by block randomization. The participants from the other two companies will be randomly allocated to Focus group (Group F) or waiting-Focus-list control group (Group C-F). Each group is expected to have about 100 participants. The real number of participants in each group will be depended on how many workers are willing to take part in the study.

Study procedure

After pre-interventions assessment, the workers with psychological distress and job strain would be invited to participant in the study. All the participants will to need to give their written informed content to the research team. The researchers will assign the participants into the intervention groups or control groups by block randomization. After randomization, two well-trained research assistants will notify each participant about the group he or she will take part. The intervention groups will be led by the relevant experts. The contents of the interventions are list below. All the participants would be measured five times with an interval of four weeks between measurements. These measurements will be taken at pre-intervention (T1), at mid-intervention (T2), at the completion of intervention (T3), four weeks after intervention (T4), and eight weeks after intervention (T5). The research assistants will distribute and collect the questionnaire.

**Intervention groups**

| Week | Measure | Content of MBI | Content of Focus group |
| --- | --- | --- | --- |
| 0 | V | N/A | N/A |
|  |  | Introduction to MBI group  Body scan  Mindful eating | Introduction to Focus group  Discussion on the definition and content of job stain |
|  |  | The association between thought and feeling  Sitting meditation  Discussion on homework exercises | Discussion on the work stressors  Finding out the possible solutions for job strain |
|  |  | Visual and sound meditation  Walking meditation  Discussion on homework exercises | Developing available strategies to enhance workers’ mental health  Redesigning work content  Inviting the management into groups, if applicable |
|  | V | Mountain meditation  Yoga  Discussion on homework exercises | Developing available strategies to enhance workers’ mental health  Redesigning work content  Generating new ideas about stress management  Inviting the management into groups, if applicable |
|  |  | Dealing with difficult situations with mindfulness  Breathing exercise  Discussion on homework exercises | Developing available strategies to enhance workers’ mental health  Generating new ideas about stress management  Inviting the management into groups, if applicable |
|  |  | Loving-kindness meditation  One minute or three minutes of mindful breathing  Discussion on homework exercises | Conducing the program  Monitoring the process |
|  |  | Choiceless awareness meditation  Breathing exercise  Discussion on homework exercises | Conducing the program  Monitoring the process |
|  | V | Integration  Mindfulness of daily activities  Building a better relationship with yourself | Integration and reviewing |
| 12 | V | N/A | N/A |
| 16 | V | N/A | N/A |

Measures

The survey questionnaire will compose of five parts: personal data, Chinese Health Questionnaire, prolonged fatigue, Job Content Questionnaire, and Perceived stress.

**Chinese Health Questionnaire.** Psychological distress will be measured by the Chinese Health Questionnaire (CHQ-12), a well-validated instrument (Cheng & Williams, 1986; Chen et al., 2000). The CHQ-12 was adapted from the General Health Questionnaire (Goldberg, 1978) with culturally relevant modification. The twelve items in the questionnaire, including questions pertaining to depression, anxiety, sleep disturbance, somatic concerns, and interpersonal difficulties, were on a four-point Likert scale. A higher score indicated a high level of psychological distress.

**Checklist Individual Strength questionnaire.** The Checklist Individual Strength questionnaire (CIS), which confirmed discriminant validity and convergent validity, will be used to measure prolonged fatigue (Beurskens et al., 2000). The CIS was developed to assess the extent of the subjective fatigue, reduction in motivation, reduction in activity, and reduction in concentration. The CIS consists of 20 items on a 7-point Likert scale ranging from 1 to 7. A higher score represented a higher level of prolonged fatigue.

**Job Content Questionnaire.** The Job Content Questionnaire (JCQ) (Karasek, 1985) is a commonly used questionnaire to assess job control (including 6 items pertaining to skill discretion and 3 items pertaining to decision authority), job demands (5 items), and workplace social support (8 items). The translated Chinese version of the JCQ was reported to have acceptable psychometric properties and will be adopted in the study. For the purpose of the study, only the subscales of job control and job demands will be obtained. A higher-sum score represents a higher level of job control or job demands.

**Perceived stress.** The 10-item Perceived Stress Scale (PSS-10) developed by Cohen and his colleagues will be adopted to measure a global level of perceived stress (Cohen, et al, 1983). Participants will be asked to respond to each question on a 5-point Likert scale ranging from 0 (never) to 4 (very often). A higher total score indicates a higher level of uncontrollable, unpredictable, and overwhelming feelings.

Statistical analysis

The percentages of personal characteristics of the participants will be presented. The comparisons between Group M and Group C-M and between Group F and Group C-F will be conducted separately using the chi-square test or Student’s t-test. The general linear model will incorporate repeated measures: a two-way mixed design ANOVA will be performed individually to determine the interactive effects and then the simple main effects of each intervention and the five continuous time points for the measured dependent variables. In addition, in order to explore the influences of personal characteristics on the outcomes of each intervention with time respectively, the interaction and simple main effects of time by gender, age, education, and occupation on the measured dependent variables will be analyzed as well.

**Reference**

- Beurskens AJ. Bültmann U. Kant I. Vercoulen JH. Bleijenberg G. Swaen GM. Fatigue among working people: validity of a questionnaire measure. Occupational & Environmental Medicine. 57(5):353-7, 2000.
- Carlson LE, Speca M, Patel KD, Goodey E. Mindfulness-based stress reduction in relation to quality of life, mood, symptoms of stress and levels of cortisol, dehydroepiandrosterone sulfate (DHEAS) and melatonin in breast and prostate cancer outpatients. Psychoneuroendocrinology 29(4):448-74, 2004.
- Chen CS, Tsang HY, Chong MY, Tang TC. Validation of the Chinese Health Questionnaire (CHQ-12) in community elders. Kaohsiung J Med Sci 2000; 16: 559-65.
- Cheng Y, Luh WM, Guo YL (2003) Reliability and validity of the Chinese version of the Job Content Questionnaire in Taiwanese workers. Int J Behav Med 10: 15-30.
- Cheng, T.A. & Williams, P. (1986). The design and development of a screening questionnaire (CHQ) for use in community studies of mental disorders in Taiwan. *Psychol Med, 16*, 415-422.
- Cohen, S., Kamarck, T., Mermelstein, R. (1983). A global measure of perceived stress. Journal of Health and Social Behavior, 24, 385-396.
- Giga SB, Cooper CL, Faragher B. The development of a framework for a comprehensive approach to stress management interventions at work. Int J Stress Manage. 10:280-296, 2003.
- Goldberg, D.P. (1978). *The manual of the general health questionnaire.* London: NFER-NELSON Publish.
- Huang SL, Lee HS, Li RH, Lai YM, Chen AL, et al. (in submission) Differences in health complaints among Taiwanese workers in different occupational categories. J Occup Health.
- Hwu YH (2008). Qualitative Research: theory, method and study. Taipei, TW. (in Chinese)
- Kabat-Zinn J (2005) Coming to our senses. New York: Hyperion.
- Karasek RA (1985) Job content questionnaire and user’s guide. Lowell: University of Massachusetts.
- Kawakami N, Araki S, Kawashima M, Masumoto T & Hayashi T. Effects of work-related stress reduction on depressive symptoms among Japanese blue-collar workers. Scand J Work Environ Health. 23(1):54-9, 1997.
- Krueger, R.A., & Casey, M.A. (2009). Focus groups: A practical guide for applied research (4th Ed.).Thousand Oaks, CA: Sage Publications.
- Marchand A, Demers A, Durand P (2005) Do occupation and work conditions really matter? A longitudinal analysis of psychological distress experiences among Canadian workers. Sociol Health Illn 27: 602-627.
- Mino Y, Babazono A, Tsuda T & Yasuda N. Can stress management at the workplace prevent depression? A randomized controlled trial. Psychother Psychosom. 75(3):177-82, 2006.
- Peters KK & Carlson JG. Worksite stress management with high-risk maintenance workers: a controlled study. Int J Stress Manage. 6(1):21-44, 1999.
- Robinson FP, Mathews HL, Witek-Janusek L. Psycho-endocrine-immune response to mindfulness-based stress reduction in individuals infected with the human immunodeficiency virus: a quasi-experimental study. J.Altern.Complement Med. 9(5):683-94, 2003.
- Witek-Janusek L, Albuquerque K, Chroniak KR, Chroniak C, Durazo-Arvizu R, Mathews HL. Effect of mindfulness based stress reduction on immune function, quality of life and coping in women newly diagnosed with early stage breast cancer. Brain Behav.Immun. 22(6):969-81, 2008.

Attachment1，AF01-010/04.3

Initial Review Protocol Application Form

| Protocol No. | |  | IRB No. | | | | | 110606 | | |
| --- | --- | --- | --- | --- | --- | --- | --- | --- | --- | --- |
|  |  |  | Receive date (filled by IRB) | | | | | (YY/MM/DD) | | |
| Protocol title | | Effects of mindfulness based stress reduction and focus group on dealing with job stress and emotional disturbance: a longitudinal study with randomized quasi experimental design. | | | | | | | | |
| Members | | Chinese name | | English name | | | Phone/extension | | | e-mail |
| Principal investigator | | 湯豐誠 | | Feng-Cheng Tang | | | 04-7238595#4131 | | | 106159@cch.org.tw |
| Coordinate investigator | | 黃淑玲 | | Shu-Ling Huang | | | 04-24730022#12301 | | | shuling@csmu.edu.tw |
| Researchers | | 李仁豪 | | Ren-Hau Li | | | 04-24730022 #11351 | | | davidrhlee@yahoo.com.tw |
| Researchers | | 黃鳳英 | | Feng-Ying Huang | | | 02-66396688# 55728 | | | fayin66h@gmail.com |
| Researchers | | 張梅香 | | Mei-Hsiang Chang | | | 0981-680353 | | | home2781454@yahoo.com.tw |
| Researchers  (add or delete column when necessary) | | 莊琇菱 | | Hsiu-Ling Chuang | | | 04-7238595/4131 | | | 396477@cch.org.tw |
| Contact person  ■ In hospital  □ Outside hospital | | 湯豐誠 | | Feng-Cheng Tang | | | 04-7238595#4131 | | | 106159@cch.org.tw |
| Subjects insurance | | ■ No | | - Yes： | | | | | | |
| Trial funding sources | | - No funding - Sponsor/pharmacetical company： - CCH - Other hospital： - School： - Association： - National Science Council - National Health Research Institute   ■ Other government departments： Health Promotion Administration, Ministry of Health and Welfare   - Other： | | | | | | | | |
| Related document | | Version/Date | | | | | | | | |
| Protocol | | 1.0 / 201105016 | | | | | | | | |
| Informed consent form | | 1.0 / 201105016 | | | | | | | | |
| Case report form | | N/A | | | | | | | | |
| Investigational brochure | | N/A | | | | | | | | |
| Advertisement | | N/A | | | | | | | | |
| (add or delete column when necessary) | |  | | | | | | | | |
| Study period | | __1_ year_0__month | | | | | | | | |
| Apply status | | ■ Expedited review | | | | - Full board review | | | | |
|  |  | - This protocol is similar to other protocol approved by IRB, IRB No.： - For other IRB review   ■ No  □ Yes  □with review results, please attach： 。  □Other IRB has approved, please attach approval letter。 | | | | | | | | |
| Executing institute  (Location) | | - CCH - CCH branch： - other domestic institute： - other participating country：   ■ Other：Four companies. The companies having good research cooperation with the PI will be chosen.  If the protocol led by CCH principal investigator, does it need to set up multi-center communication channel?  □Yes ■No □Do not apply | | | | | | | | |
| Research type | | - Drug registration (Please attach investigational medicinal product/ vaccine introduction) - Drug academic research (Please attach investigational medicinal product/vaccine introduction)) - Post marketing surveillance (Please attach investigational medicinal product/vaccine introduction)) - Medical devices or medical devices with new medical techniques (Please attach medical devices or medical devices with new medical techniques introduction) - Medical techniques (Medical technique introduction) - Generic drug bioavailability, bioequivalence - Genetic research (including genetic test, transgenic, stem cells, genetic recombinant ) - Epidemiological studies - Social behavioral sciences   ■ Other：_Behavior treatment_______ | | | | | | | | |
| Research model | | ■ Intervention research   - 1. Research purpose：   □Treatment  □Prevent  □Diagnose  □Supportive treatment  □Screening  □Health policy  □Basic medical  ■other: Enhancing mental health   - 1. Random distribution：   □Single group  ■random distribution  □non-random distribution   - 1. Blinding test：   ■Open  □single blind  □double blind  □triple blind   - 1. Comparator：   □Placebo  □Dosage comparison  □other effective treatment  □Previous information  □None  ■other waiting-list control group   - 1. Intervention model：   □single group  □double group parallel  □double group intersect  ■more than 3 groups   - 1. End point：   □safety  ■efficacy  □Bioequivalence  □Bioavailability  □Pharmacokinetics  □Pharmacodynamic   - Observative research  1. Observation model：   □Cohort Study  □Case control study  □Case study  □case crossover study  □ecological or social research  □family research  □other   1. data collection period：   □Retrospective  □Prospective  □cross-sectional study | | | | | | | | |
| Measure tools | | ■ Questionnaire  □Record (including interview, recording, video tape recording) | | | | | | | | |
| Retention of biological samples | | - Non DNA biological samples (e.g. fix structure, serum…) - Retain DNA biological samples (e.g. frozen structure…)   ■ No biological samples/none retain biological samples | | | | | | | | |
| Test phase | | - phase I：The safety research in understanding the toxicity of the drug with healthy volunteer subjects. The subjects are healthy volunteers. - phase II：The initial efficacy observation in understanding the efficacy of the drug. The subjects are patients. - phase III： The complete efficacy evaluation in order to confirm the efficacy and the safety. The subjects are patients and comparator groups. - Phase IIIa：Haven’t passed the review of the competent authority. - Phase IIIb： Passed the review of the competent authority. - phase IV：post marketing surveillance (PMS) to see if there is any adverse reaction for long-term tracking. - Other： 。 | | | | | | | | |
| Sample quantity | | - Global _______ - Domestic _400__ | | | | - CCH：_______ - Other：___(please specify the institute and the number of people) | | | | |
| Trial subjects | | - Age：___18_____～___65_____(years old) | | | | | | | | |
|  |  | ■ Normal   - Patient   ■ Male  ■ Female   - Other：______ | | | □Vulnerable groups  □Prinsoner  □Pregnant women  □Infants  □Children  □Disabled people  □Other：________ | | | | | |
| Special condition | | - Intense care - Child intense care - Genetic treatment - Prosthetics | | | - Quarantine - Intravenous injection - Controlled drug - Other：________ | | | | - Surgery - CT - Gynecological supplies | |
| Inclusion criteria | | - 18 Years to 65 Years healthy employees with psychological distress and job strain - Full-time paid workers | | | | | | | | |
| Exclusion criteria | | - Age < 18y or > 65y - Part-time workers - The workers are not willing to take part in the study | | | | | | | | |
| Expected adverse event/serious adverse event | | None | | | | | | | | |
| The management of expected adverse event/ serious adverse event | | None | | | | | | | | |
| Recruit subjects | | - Media advertisement（attach the content） - TV - Radio - Newspaper - Internet - Bill board - Poster - Flyers | | | | | ■ Oral introduction   - Other________ | | | |
| Result report or publication | | Publication in academic journals or presentation in conference. | | | | | | | | |
| 1. Is there special protection in getting the informed consent of the vulnerable groups?   □Yes, specify______  □No  ■NA | | | | | | | | | | |
| 1. Is there Data safety monitoring plan, DSMP?   Explanation: except for minimal risk trials, the protocol should have DSMP. The definition of minimal risk: it means the changes or degrees of hazards caused to the body or mind is equivalent to those caused from the daily life of health subjects, conventional medicine, and psychological examination. The hazard does not increase as participating in the human trial.  □ Yes, attach DSMP protocol  ■ No | | | | | | | | | | |
| 1. Is there Data safety monitoring board, DSMB?   □Yes, attach DSMB protocol  □Comparator research: the main or secondary endpoint of the research is death and/or serious diseases.  □Randomly comparator research: the main research purpose is to evaluate the effectiveness and safety of a new intervene measure of lowering the incident rate of the serious disease or death.  □The early research of high risk intervene measures: whether random or not, e.g. unpreventable, potential lethal complications or common, concerned, preventable adverse event.  □The early phase of innovative intervene measures: the clinical safety information is limited or the previous information leads to potential serious adverse consequences.  □Complicated research or the expected accumulated data is hard to explain, or the accumulated data might affect the research design and the subject safety, especially long term research.  □The data obtained from the research proves to terminate the research. For example, the purpose of an intervene measures is to lower the incident rate or death rate of a serious disease; however, in fact it has adverse response or lack of efficacy that leads to increasing the incident rate or death rate.  □The research conducted under emergency.  □The research involving vulnerable groups.  The frequency of submitting DSMB report：_______________  ■No | | | | | | | | | | |
| 1. Whether to stop or suspend the standard treatment for trial purpose?   □Yes, specify____  □No  ■NA | | | | | | | | | | |
| 1. Whether to include subjects who cannot perform consent?   □Yes, reason______  ■No  □NA | | | | | | | | | | |
| 1. Provide medical care to the subjects during and after the trial?   □Yes  □No  ■NA | | | | | | | | | | |
| 1. Provide the subjects with trial product after the end of study?   □Yes  □No  ■NA | | | | | | | | | | |
| 1. The personnel who might get in touch with the personal information of the subjects (including the medical record and the specimen)?   Include： Principal investigator, coordinate investigator and researchers | | | | | | | | | | |
| 1. The way to keep subject information in confidentiality    - - - Subject identity code   ⬜ Use English name initials  ■ Code the research information  ■ Lock/encrypt all information  ⬜ Other： | | | | | | | | | | |
| 1. Whether the research material that does not de-link provides for specific research abroad?   □Yes, attach the warranty that the abroad research institute assure to abide by our national regulations and research material usage scope.  ■No  (Note: research material is the human specimen, nature person information, and other related data, information of the subjects.) | | | | | | | | | | |
| 1. The related procedure of obtaining informed consent:   Apply for waiver or alteration of informed consent? □No ■Yes (Please attach waive or alter informed consent checklist.)   - 1. Who explains to the subject or the legal representative about the research and obtains the consent?   □Principal investigator (including coordinate investigator)  ■Researcher  □Other doctor/research nurse (non coordinate investigator)   - 1. The language used to obtain the informed consent:   □Chinese □Taiwanese ■Chinese+ Taiwanese □Other：   - 1. Expected language understood by the subject or the legal representative   □Chinese □Taiwanese ■Chinese+ Taiwanese □Other：   - 1. The timing of obtaining the informed consent?   □Before screening  □After screening, before randomly assign  ■After screening, after randomly assign  The place of explaining the trial content? The worksites where the participant work for ；How long does it take? About 15-20 minutes | | | | | | | | | | |
| 1. Subject risk and benefit evaluation ：    1. Experiment group：   ■ Equivalent to minimal risk. (Category 1 type risk)   - Greater than minimal risk but has direct benefit to the subjects. (Category 2 type risk) - Greater than minimal risk but has no direct benefit to the subject; however, it helps understand the condition of the subjects. (Category 3 type risk) - Greater than minimal risk but has no direct benefit to the subjects; however, the research can have valuable results. (Category 4 type risk)   1. Comparator：■Yes □No(The following are optional)   ■ Equivalent to minimal risk. (Category 1 type risk)   - Greater than minimal risk but has direct benefit to the subjects. (Category 2 type risk) - Greater than minimal risk but has no direct benefit to the subject; however, it helps understand the condition of the subjects. (Category 3 type risk) - Greater than minimal risk but has no direct benefit to the subjects; however, the research can have valuable results. (Category 4 type risk) | | | | | | | | | | |
| Statement of Person Completing | I hereby acknowledge that I have filled in the above information and assure the correctness of the content. If there is any untrue or deliberate concealment, I am subject to legal liability.  Signature：_____________  Unit：Department of Occupational Medicine, Changhua Christian Hospital ____________  Date：_2011/05/06__(YYYY/MM/DD) | | | | | | | | | |
| Statement of Principal Investigator | 1. I am in charge of executing the clinical trial and have read the proposal carefully. I promise to conduct the study according to the protocol proposal approved by IRB. 2. I acknowledge and agree to abide by Declaration of Helsinki, related ethical, laws, and regulations requirements related to human trial to assure the rights, safety, personal privacy and wellfare of the subjects are protected. 3. Pursuant to the regulations, IRB has the rights to review any information related to the trial, proceed with actual site audit visit, continuing review the process of the approved trials and that is not less than one time a year. I acknowledge that I would cooperate with it and submit the interim report in time. 4. If there is any major condition influencing the conduct of study or increase the risks of the subject, I should report it to IRB in writing. 5. If any unexpected adverse events or serious adverse drug reaction happen to the subjects, I should immediately inform IRB and the competent authority and provide detailed written information. 6. Besides to avoid the subjects being harmed in time, I do not deviate or modify the protocol before getting the approval of IRB. 7. If the trial is completed or terminated earlier, I should propose the sumary of trail result to IRB.   Signature of Principal investigator：_____________  Unit：Department of Occupational Medicine, Changhua Christian Hospital _  Date： 2011/05/06 (YYYY/MM/DD) | | | | | | | | | |
