# Supplementary material for: The Potential for Mindfulness-Based Intervention in Workplace Mental Health Promotion: Results of a Randomized Controlled Trial
Source: PLoS One. 2015 Sep 14;10(9):e0138089. doi: 10.1371/journal.pone.0138089 (PMC4569475; doi:10.1371/journal.pone.0138089)
Supplement: S3 Protocol — (DOC) [file pone.0138089.s005.doc]

Overview of the MBI program

| Session | Content | Home practice |
| --- | --- | --- |
| Session One | - Brief introduction to program and filling out pre-intervention questionnaire - Setting up the guidelines for participation: confidentiality, respecting others’ experiences, non-judging, self-caring, communicating with leader or facilitator - Giving opportunities to participants to introduce themselves and what bring them here - Raisin-eating exercise - Practices on mindful breathing and body scan | - Body Scan (40 minutes) and recording per day - Eating mindfully or doing any daily activity mindfully as much as they can |
| Session Two | - Guided body scan - Standing Yoga - Group Discussion on the experiences of practicing body scan and eating mindfully - Introduce sitting meditation with awareness of breathing as primary object of attention - Closing session with a short body scan | - Body Scan (40 minutes) and recording per day - Mindful breathing practice (10-15 minutes) - Mindfulness on daily routine activities - Filling out Pleasant Events Calendar during the week |
| Session Three | - Introducing walking meditation. - Group Discussion on challenges and insights from the experiences of the in-session and home assigned practices - Going through the pleasant events calendar - Sitting meditation with awareness of breathing - Closing session with a short Yoga | - Body scan (40 minutes) 3 times in the week - Sitting meditation (30 minutes) with awareness of breathing (10 minutes) for the other 4 days in the week - Filling out Unpleasant Events Calendar during the week |
| Session Four | - Introducing various Yoga postures - Sitting meditation with awareness of sound, and breathing - Focusing on mindful ways to observe physical sensations, emotions, and thoughts as they arise - Group discussion exploring the in-session and home assigned sitting meditation - Going through the unpleasant events calendar - Closing the session with a short guided Loving-Kindness meditation | - Sitting meditation 30 minutes per day with attention on breathing, physical sensations, sounds, and awareness of the whole body - Yoga postures for 15 minutes - Using 3 minutes mindfulness practice while encountering difficult situations in daily life |
| Session Five | - Yoga practice - Group discussion on habitual behavioral patterns, thoughts and emotions, that are associated with experiences of feeling stuck, blocking, numbing, and shutting off in daily life - Sitting meditation with awareness of breath, body, sounds, emotions, thoughts, as “events” in consciousness - Closing the session with a short guided Loving-Kindness meditation | - New sitting meditation with awareness of breath, sounds, emotions, and thoughts (40 minutes) and recording per day - Guided Loving-Kindness meditation (15 minutes) and recording per day - 3 minutes mindfulness practice when encountering difficult situations during the week |
| Session Six | - Various Yoga postures - Sitting meditation with awareness of breathing - Moving from meditation to guided reflection on what mindfulness practice brings into lives and how mindfulness practice helps to deal with stress and difficulties on communication with others - Introducing Mountain meditation - Closing the session with a short guided Loving-Kindness meditation | - Mountain Meditation or Body Scan (40 minutes) per day - Yoga postures for 15 minutes per day - 3 minutes mindfulness practice when encountering difficult situations during the week |
| Session Seven | - Brief sitting meditation in silence - Introducing Choice-less Awareness Meditation - Yoga Practice - Group Discussion on mindfulness practice and experiences of dealing with difficulties on communication - Closing the session with a short awareness of breathing | - Choiceless Awareness Meditation or silent meditation (40 minutes) per day - Guided Loving-Kindness meditation recording (15 minutes) per day - 3 minutes mindfulness practice when encountering difficult situations during the week |
| Session Eight | - Body Scan - Yoga stretching - Sitting meditation - Group dialogue and discussion: Reviewing the entire course and focusing briefly on salient experience and features - Discussing how to remind themselves to keep practicing - Closing the group and filling out the post-intervention questionnaires | - Body scan or any recording as they wish |
